# Sydenham Society of London

**Published:** 1852-02

**Authors:** 


### SYDENHAM SOCIETY OF LONDON.

The first volume of the Society's works for the year 1851–'2, has reached this country. It embraces the "Principles of Physiology" of Unzer; and a "Dissertation on the Functions of the Nervous System," by Prochaska; translated and edited by Dr. Thomas Laycock. It is hoped, that another volume of the Annals of Influenza has appeared by this time in London. The third volume is in press, the fourth of Rokitansky.

